# Temperature modulates dengue virus epidemic growth rates through its effects on reproduction numbers and generation intervals

**DOI:** 10.1371/journal.pntd.0005797

**Published:** 2017-07-19

**Authors:** Amir S. Siraj, Rachel J. Oidtman, John H. Huber, Moritz U. G. Kraemer, Oliver J. Brady, Michael A. Johansson, T. Alex Perkins

**Affiliations:** 1 Department of Biological Sciences and Eck Institute for Global Health, University of Notre Dame, Notre Dame, United States of America; 2 Department of Applied and Computational Mathematics and Statistics, University of Notre Dame, Notre Dame, United States of America; 3 Department of Zoology, University of Oxford, Oxford, United Kingdom; 4 Department of Pediatrics, Harvard Medical School, Boston, United States of America; 5 Department of Informatics, Boston Children’s Hospital, Boston, United States of America; 6 Centre for the Mathematical Modelling of Infectious Diseases, London School of Hygiene & Tropical Medicine, London, United Kingdom; 7 Department of Infectious Disease Epidemiology, London School of Hygiene & Tropical Medicine, London, United Kingdom; 8 Division of Vector-Borne Diseases, Centers for Disease Control and Prevention, San Juan, Puerto Rico; 9 Center for Communicable Disease Dynamics, Harvard TH Chan School of Public Health, Boston, United States of America; Institute for Disease Modeling, UNITED STATES

## Abstract

Epidemic growth rate, *r*, provides a more complete description of the potential for epidemics than the more commonly studied basic reproduction number, *R*_0_, yet the former has never been described as a function of temperature for dengue virus or other pathogens with temperature-sensitive transmission. The need to understand the drivers of epidemics of these pathogens is acute, with arthropod-borne virus epidemics becoming increasingly problematic. We addressed this need by developing temperature-dependent descriptions of the two components of *r—R*_0_ and the generation interval—to obtain a temperature-dependent description of *r*. Our results show that the generation interval is highly sensitive to temperature, decreasing twofold between 25 and 35°C and suggesting that dengue virus epidemics may accelerate as temperatures increase, not only because of more infections per generation but also because of faster generations. Under the empirical temperature relationships that we considered, we found that *r* peaked at a temperature threshold that was robust to uncertainty in model parameters that do not depend on temperature. Although the precise value of this temperature threshold could be refined following future studies of empirical temperature relationships, the framework we present for identifying such temperature thresholds offers a new way to classify regions in which dengue virus epidemic intensity could either increase or decrease under future climate change.

## Author summary

Recurrent, rapidly intensifying epidemics of dengue–the world’s most prevalent mosquito-borne viral disease–pose a challenge to healthcare systems throughout the tropical and subtropical world. An acute disease that tends to respond well to proper treatment, the sometimes intense nature of dengue epidemics has been known to overwhelm healthcare systems and elevate the morbidity and mortality of patients left without adequate medical treatment under peak epidemic conditions. Here, we quantify the temperature dependence of dengue epidemic intensity by quantifying two distinct determinants of epidemic growth rate: the average number of secondary infections arising from each primary infection and the average time between successive infections in humans. Our results show that the time between successive infections in humans decreases steadily with increasing temperatures, whereas the average number of secondary infections peaks at intermediate temperatures. Altogether, this suggests a peak temperature for dengue epidemic intensity. Applying this result to global temperature projections under future climate change scenarios suggests that dengue epidemics in many regions of the world could become more intense under future temperature increases.

## Introduction

Dengue virus (DENV) is a mosquito-borne pathogen that infects hundreds of millions of people each year across as many as 128 countries [1]. Along with numerous other arthropod-borne viruses (arboviruses), including chikungunya and Zika viruses [2,3], DENV causes epidemics with considerable public health impact. Rapidly growing, intense epidemics can overwhelm healthcare systems [4], leaving those infected without adequate medical treatment and with a significantly elevated risk of mortality to a disease that is seldom fatal when proper treatment is available [5].

A number of factors can lead to variability in the frequency and severity of arbovirus epidemics, including importation probability [6], host susceptibility [7], and climatic conditions [8]. In particular, temperature is known to be a major driver of spatial and temporal variability in arbovirus transmission, as indicated by empirical studies of relationships between temperature and several epidemiologically important vector and pathogen traits, including mosquito lifespan [9–11], incubation time of the pathogen in the mosquito [9,10,12], the rate at which mosquitoes engage in blood feeding [9,13], and mosquito density [14].

Analyses of the effects of temperature on vector-borne pathogen transmission have focused primarily on the basic reproduction number *R*_0_ through the effects of temperature on the aforementioned vector and pathogen traits [11,15,16]. Defined as the average number of secondary infections arising from a primary infection in a fully susceptible population, *R*_0_ is a fundamentally important epidemiological quantity, because it is informative about the conditions under which a pathogen can invade, or be eliminated from, a host population. The generation interval, which is the period of time separating sequential infections, is the temporal analogue of *R*_0_. Through a fundamental mathematical relationship [17], *R*_0_ and the generation interval are related to the epidemic growth rate *r*, which is defined as the per capita change in incidence per unit time and characterizes the dynamics of early-stage epidemic growth in a susceptible population. Because the relationship between *r* and temperature has never been characterized for arboviruses, there is little scientific basis for understanding how epidemic growth rates may be related to temperature.

Our goal was to quantify the effects of temperature on DENV epidemic growth rates by first establishing a probabilistic description of DENV generation intervals as a function of temperature. We then combined our generation interval calculations with a temperature-dependent formulation of the basic reproduction number, *R*_0_, and solved for the epidemic growth rate *r* as a function of temperature. This new capability to calculate *r* as a function of temperature allowed us to identify temperature ranges that maximize *r* and to classify regions by their potential for increasing or decreasing epidemic growth rates based on their current and future temperatures. Our results and the accompanying code are made freely available online at https://github.com/asiraj-nd/arbotemp to facilitate the incorporation of temperature-dependent descriptions of these quantities into future studies.

## Materials and methods

We first describe our formulation of each of three major metrics of mosquito-borne pathogen transmission: the generation interval, the basic reproduction number *R*_0_, and the epidemic growth rate *r*. The first two are calculated a priori as a function of many of the same temperature-dependent parameters, whereas the third is derived from the first two using a fundamental mathematical relationship among all three. We then describe analyses that we performed to evaluate the epidemiological significance of these three different measures of how temperature impacts dengue virus transmission.

### Generation interval

**Fig 1:**
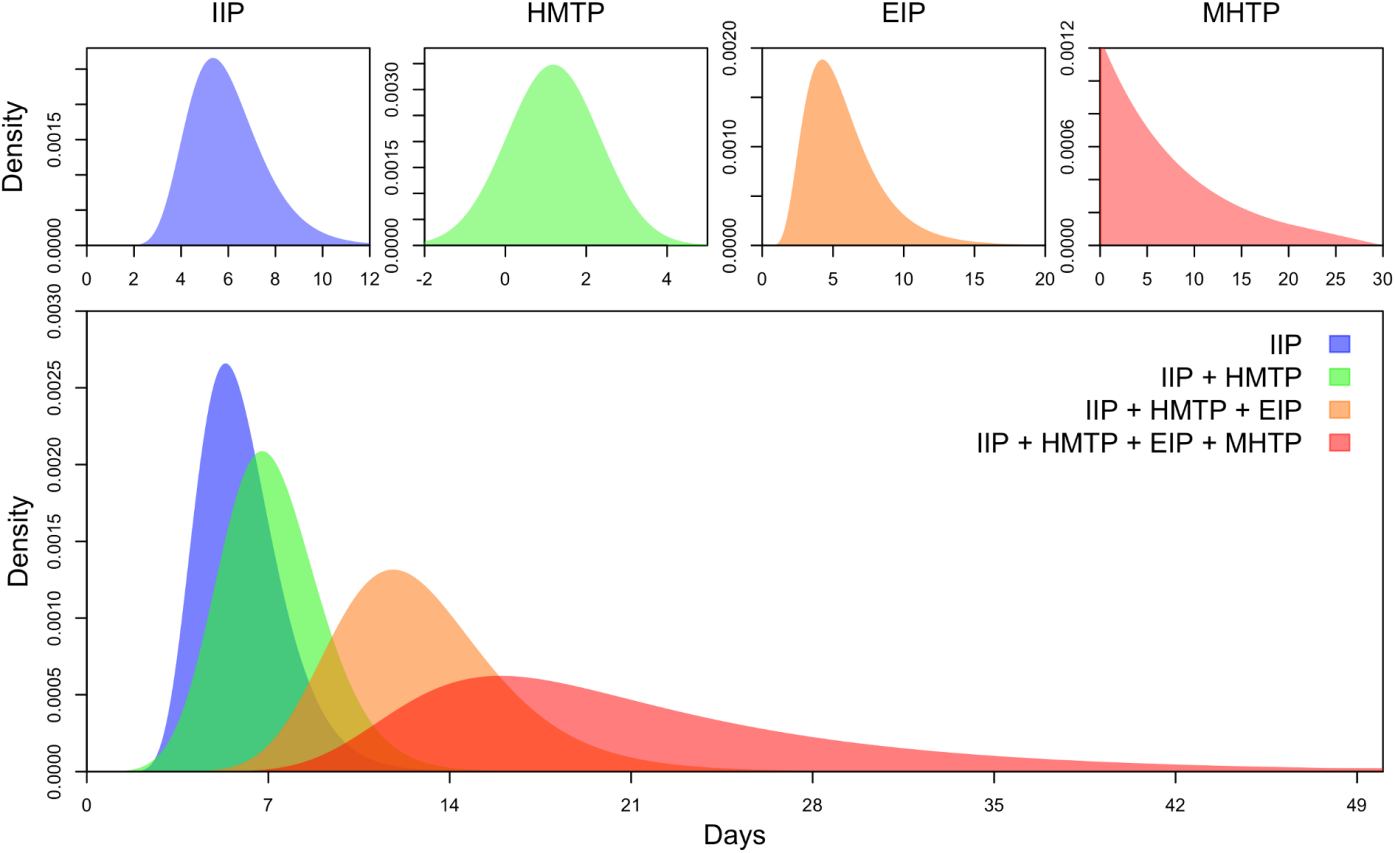
Random variables associated with components of the transmission cycle (top) and their successive sums (bottom). On the top, the intrinsic incubation (IIP), human-to-mosquito transmission period (HMTP), extrinsic incubation period (EIP), and mosquito-to-human transmission period (MHTP) are shown from left to right, with the latter two parameterized for a temperature of 30°C. On the bottom, random variables for the elapsed time between inoculation of the primary infection and each event in the transmission cycle is shown in successive order from left to right.

We define the generation interval as the elapsed time between a primary human infection and a secondary human infection deriving from that primary human infection via two bites from the same individual mosquito [18]. To derive a quantitative, probabilistic description of the generation interval for dengue, we adapted an existing framework that defines the generation interval as a sum of random variables for each of four sequential, constituent phases of the transmission cycle [19]. Similar to a recent analysis for *Plasmodium falciparum* malaria [20], we furthermore quantified each of these phases of the transmission cycle as dependent on temperature (Fig 1). Following Huber et al. [20], we defined these phases as: (1) the intrinsic incubation period (IIP); (2) the period between onset of symptoms in humans and subsequent transmission to mosquitoes (human-to-mosquito transmission period, HMTP); (3) the extrinsic incubation period (EIP); and (4) the period between a mosquito becoming infectious and subsequent transmission to humans (mosquito-to-human transmission period, MHTP) (Fig 1). Below, we describe the derivation and parameterization of each of these phases of the transmission cycle as four independent random variables based on available data [13,21,22]. To obtain a single random variable describing the generation interval as a whole, we took the sum of the four constituent random variables in Fig 1 by applying the convolution theorem, which involves taking the inverse Fourier transform of the product of the Fourier transforms of each random variable [23].

#### Intrinsic incubation period (IIP)

Defining the intrinsic incubation period (IIP) as “the time between a human being infected and the onset of symptoms,” Chan and Johansson [12] fitted time-to-event models to 204 observations from 35 studies. They concluded that differences in the IIP across serotypes were indistinguishable and that the IIP was best described by a lognormal distribution with mean 5.97 days and standard deviation 1.64 days. Given their comprehensive treatment of data from a broad range of studies, we adopted this best-fit lognormal distribution in our analysis (Fig 1, IIP).

#### Human-to-mosquito transmission period (HMTP)

We define the human-to-mosquito transmission period (HMTP) as the entirety of the elapsed time between the conclusion of the IIP and when a susceptible mosquito becomes infected. This depends on both the duration of infectiousness and the timing of a person’s infectiousness over the course of their infection. We based our estimates of HMTP on studies in which people were experimentally infected and their infectiousness to mosquitoes assessed over time. These data have been re-analyzed and compiled on a daily basis relative to onset of fever [22], which is compatible with the endpoint of the IIP described above. To obtain a probabilistic estimate of the period of transmission from a human host to a mosquito vector, we fitted a normal probability density function multiplied by a constant scaling factor to the data presented by Nishiura and Halstead [22] using numerical likelihood maximization (Fig 1, HMTP) and normalized it to yield a description of the probability that an infected mosquito acquired the infection on day *t* relative to the onset of symptoms in its human blood-meal host. This interpretation assumes that mosquito densities and biting rates are constant over the human infectious period.

#### Extrinsic incubation period (EIP)

Chan and Johansson [12] proposed a lognormal distribution for the length of the extrinsic incubation period with the scale parameter a function of temperature, *e*^2.9−0.08 *T*^, and the shape parameter a constant, 4.9. Given their comprehensive treatment of data from a collection of 38 studies, we adopted this best-fit lognormal distribution in our analysis (Fig 1, EIP).

#### Mosquito-to-human transmission period (MHTP)

This period covers the time between a mosquito becoming infectious (at the end of the EIP) and it biting a susceptible human host and causing an infection. A probability distribution describing the length of this period could potentially depend on several variables, including mosquito biting and mortality schedules. Although there is some evidence for age-dependent mortality in wild *Aedes* mosquitoes [24], incorporating age-dependent effects in a general model of the mosquito-to-human transmission period would depend not only on knowledge of age-dependent mortality but also age-dependent exposure to infection [25]. Because of the extensive variability of these relationships across different ecological settings [26] and the difficulty of quantifying these effects based on available data [13], we restricted our analysis to age-independent mortality, which is a common assumption of dengue transmission models [27]. Conditional on a mosquito surviving the EIP, its lifespan thenceforth can be described as an exponential random variable parameterized by a mean, age-independent daily mortality rate. Following Perkins et al. [28], we used the temperature- and age-dependent model by Brady et al. [11], to which we added an additional rate of extrinsic mortality (0.089 d^-1^) to match an empirical estimate of overall daily mortality of 0.115 carried out in an experiment in which temperatures ranged 20–34°C [21] (Fig 1, MHTP). To match our simplifying assumption of age-independent mortality, we parameterized the mortality rate in our model to yield an average lifespan consistent with that of the model by Brady et al. [11] for a given temperature. Although our primary results were calculated based on functions of mean temperature, we also explored the effect of diurnal temperature fluctuations using diurnally varying hazards for mosquito mortality and EIP (see S2 Appendix).

### Basic reproduction number

The basic reproduction number (*R*_0_) is defined as the average number of secondary infections in humans originating from a single primary human infection introduced into a fully susceptible population. We used the formal definition of *R*_0_ for mosquito-borne pathogens based on a set of classic “Ross-Macdonald” assumptions [29], which takes the temperature-dependent form

$$R_{0}(T)=\frac{m(T)bc{a(T)}^{2}e^{-\mu (T)n(T)}}{\mu (T)\;\gamma },$$ (1)

where *m*(*T*) is the mosquito-to-human ratio as a function of temperature *T*, *μ*(*T*) is the mean daily mortality rate of adult mosquitoes at temperature *T*, *b* and *c* are human-to-mosquito and mosquito-to-human infection probabilities, *a*(*T*) is the mosquito biting rate as a function of temperature, 1/γ is the average duration of infectiousness in humans, and *n*(*T*) is the mean extrinsic incubation period at temperature *T*. We note that the mean daily mortality rate of adult mosquitoes, *μ*(*T*), is the inverse of the mean for the MHTP distribution used in obtaining the generation interval distribution, while the mean extrinsic incubation period, *n*(*T*), is the mean for the EIP distribution, also used in obtaining the generation interval distribution.

Our parameterization of the ratio *c*/γ equaled the integral of the non-normalized HMTP curve describing the infectiousness of humans to mosquitoes over time [22], as noted in the section describing the generation interval. The parameter *b* did not appear in our description of the generation interval, because it affects only the magnitude of transmission (i.e., *R*_0_) rather than its timing (i.e., generation interval). This parameter is poorly understood empirically, so we chose a value of *b* = 0.4 consistent with a previous model [30]. We described biting rate *a* as a function of temperature *T* (i.e., *a*(*T*)) using two temperature-dependent estimates based on the average duration of the *Ae*. *aegypti* gonotrophic cycle [9,31], similar to how gonotrophic period was incorporated into the generation interval. This process involved weighting the temperature-dependent length of the first cycle and the temperature-dependent length of each subsequent cycle based on the probability of the mosquito surviving to a given number of cycles (see S1 Appendix for mathematical derivation). To capture one potential effect of temperature on the ratio of mosquitoes to humans *m*, we assumed that *m*(*T*) = λ / *μ*(*T*) consistent with equilibrium assumptions of a mosquito population with adult mortality rate *μ*(*T*) and constant parameter λ, which is the ratio of the daily rate of adult female mosquito emergence and the number of humans subject to feeding by the mosquitoes represented by *m*(*T*) [32]. Because values of λ are highly variable in space and time for reasons other than temperature variation, we examined the sensitivity of the value of λ across a range of values 0.0–0.5. We arrived at 0.5 as an upper limit for λ by dividing an upper limit for *R*_0_ based on independent estimates (maximum of 7.8 [33]) by all other terms on the right-hand side of Eq 1 (19.73 at 32.5°C). This is equivalent to assuming that one new adult female mosquito emerges from larval habitats every other day for each human at risk of biting within a given population.

To account for uncertainty associated with values of *R*_0_ that we calculated, we generated 1,000 Monte Carlo samples from the uncertainty distributions of each model parameter as described in each of the references [9,12,13] in which those parameters were originally described. For *μ*(*T*) and *n*(*T*), we took random draws of their parameters consistent with published descriptions of uncertainty in the parameters of these functions from their original sources [13,14]. For *a*(*T*), we used nonlinear least-squares estimates of the first gonotrophic period’s *ρ* parameter in the model by Focks et al. [9] by refitting it to their data, resulting in mean 8.83x10^-3^ and standard deviation 3.8x10^-4^. We assumed similar uncertainties (standard deviation) around the *ρ* parameter for the second gonotrophic period proposed by Otero et al. [31]. We then took random draws from normal distributions describing uncertainties in these two parameters and weighted the resulting two temperature-dependent biting rates (inverses of the gonotrophic periods) according to the probability of the mosquito surviving to a given number of gonotrophic cycles, as described in S1 Appendix. A summary of parameters and their default values is available in S4 Table.

### Epidemic growth rate

Given temperature-dependent formulations of *R*_0_(*T*) and the DENV generation interval *g*(*t*) described above, we solved for the corresponding epidemic growth rate *r*(*T*) as a function of temperature by applying the result

$$\frac{1}{R_{0}(T)}=\int_{0}^{\infty }e^{-r(T)t}g(t)dt$$ (2)

from Wallinga and Lipsitch [17]. Although this does not yield an explicit relationship between *r* and *T* that can be probed analytically, it does provide a way of numerically characterizing the impacts of temperature on *r*. We further note that this approximation of *r*(*T*) assumes a fully susceptible, well-mixed population of mosquitoes and hosts.

### Analyses

We first derived a formulation of the generation interval for dengue, stochastic variability therein, and its dependence on temperature based on the assumptions described above. We then performed analyses of the relationship between temperature and *r*, including identification of the temperature that maximizes *r* and how incremental changes in *r* driven by changes in temperature can be attributed to distinct contributions from changes in *R*_0_ versus changes in the generation interval. For comparison with our detailed formulation of the epidemic growth rate *r*, we examined two approximations of the generation interval commonly used in transmission models: a fixed-length generation interval and an exponentially distributed generation interval. For each, we considered two formulations: one with a mean generation interval of 16 days [34] and one with temperature-dependent mean generation interval as calculated using our method.

We next considered how average monthly temperature data at 5 km x 5 km resolution for each month of the year based on historical records (average for 1950–2000) [35] may change epidemic growth rates under climate change scenarios. For this analysis, we used three different scenarios for mean temperature in 2050 (average for 2040–2060) corresponding to Representative Concentration Pathways (RCPs) that describe a set of alternative trajectories for the atmospheric concentration of key greenhouse gases: RCP 8.5, high greenhouse gas concentration scenario; RCP 6.0, medium baseline (or high mitigation) scenario; and RCP 4.5, intermediate mitigation scenario [36]. We obtained gridded population estimates for the year 2000 from the Global Rural/Urban Mapping Project [37] and for 2050 by projecting values from 2000 onward according to medium-fertility population projections for each country [38]. We excluded regions from this analysis where *Ae*. *aegypti* occurrence probabilities fall below 0.8, a threshold value that separates two distinct modes of local occurrence probabilities globally [39,40].

Potential for diurnal temperature fluctuations to influence DENV transmission has been suggested by temperature effects on extrinsic incubation period (EIP) and mosquito survival [10]. We examined potential effects of diurnal temperature fluctuations on the generation interval, basic reproduction number *R*_0_, and epidemic growth rate *r* by introducing an 8°C diurnal temperature range (DTR) around all mean temperatures. We assumed a sinusoidal progression within the day with a decreasing exponential curve at night [9,41]. We also assumed an absolute maximum temperature for *Ae*. *aegypti* survival of 37.73°C over three consecutive hours and 40.73°C in any single hour, as well as a maximum temperature of 45.9°C in any hour of the day for DENV incubation to take place, similar to assumptions of another recent model of temperature-dependent viral transmission by *Ae*. *aegypti* mosquitoes [42].

## Results

### Characterizing temperature effects on transmission

**Fig 2:**
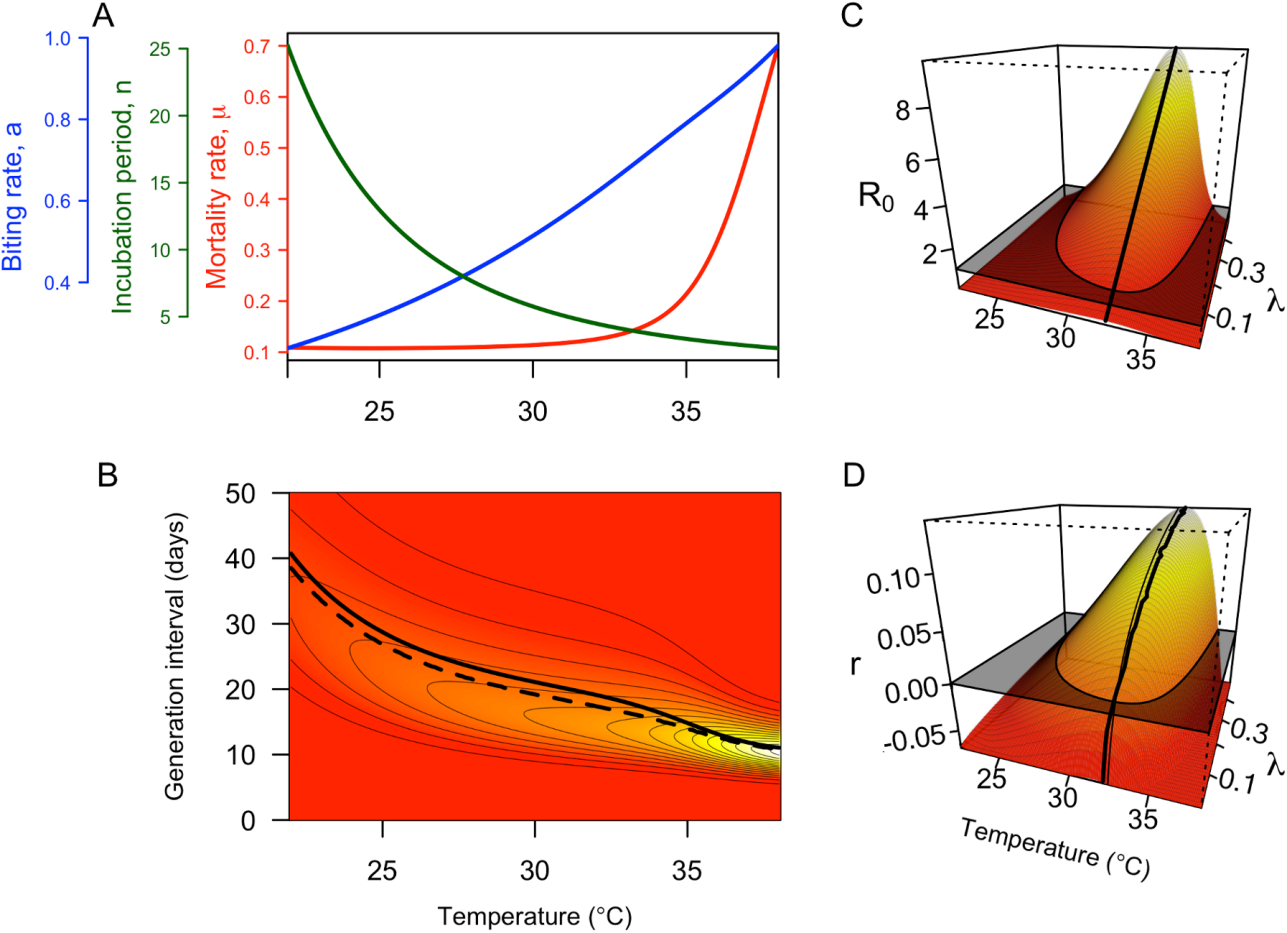
Relationships between temperature and entomological parameters (A) and epidemiological quantities (B-D). The thick solid (dashed) line in B shows the mean (median) generation interval at each temperature, and colors indicate the probability density of generation intervals at a given temperature (red to yellow = low to high). Contours show probability density values in intervals of 0.05. Colored surfaces in C and D show how temperature and mosquito emergence rate *λ* affect *R*_0_ and *r* (red to yellow = low to high), respectively. Black planes in C and D indicate the combinations of temperature and *λ* values for which *R*_0_ and *r* fall above or below threshold values (1 or 0, respectively). The thick black lines in C and D show the temperatures at which either *R*_0_ or *r* is maximized for a given value of *λ*. For comparison, the thin line in D indicates temperatures at which *R*_0_ is maximized.

We developed a probabilistic description of the DENV generation interval by sequentially summing random variables associated with each phase of the transmission cycle (Fig 1). Allowing each of these component random variables to depend on temperature (Fig 2A) resulted in a description of the generation interval that was itself strongly dependent on temperature and captured variability and uncertainty in the underlying components (Fig 2B). For example, mean generation interval halved from 30 to 15 days with a change in temperature from 25 to 35°C. Sensitivity of the mean generation interval to changes in temperature was nonlinear, with steeper changes at more extreme temperature values (Fig 2B) due to increasing steepness of the relationships between temperature and the component random variables (Fig 2A).

The basic reproduction number, *R*_0_, was also sensitive to temperature, as it includes the same temperature-dependent random variables as the generation interval. At low temperatures, increases in temperature caused a steady increase in *R*_0_ due to a shortening extrinsic incubation period and increasing biting frequency (Fig 2A and 2C). Beyond a peak temperature of 32.5°C, *R*_0_ decreased rapidly with increasing temperatures due to rapidly increasing mosquito mortality (Fig 2A and 2C). This result contrasted with a lower peak temperature (~29°C) that was obtained in our analysis (not shown in figures) under an assumption that biting rate did not depend on temperature.

**Fig 3:**
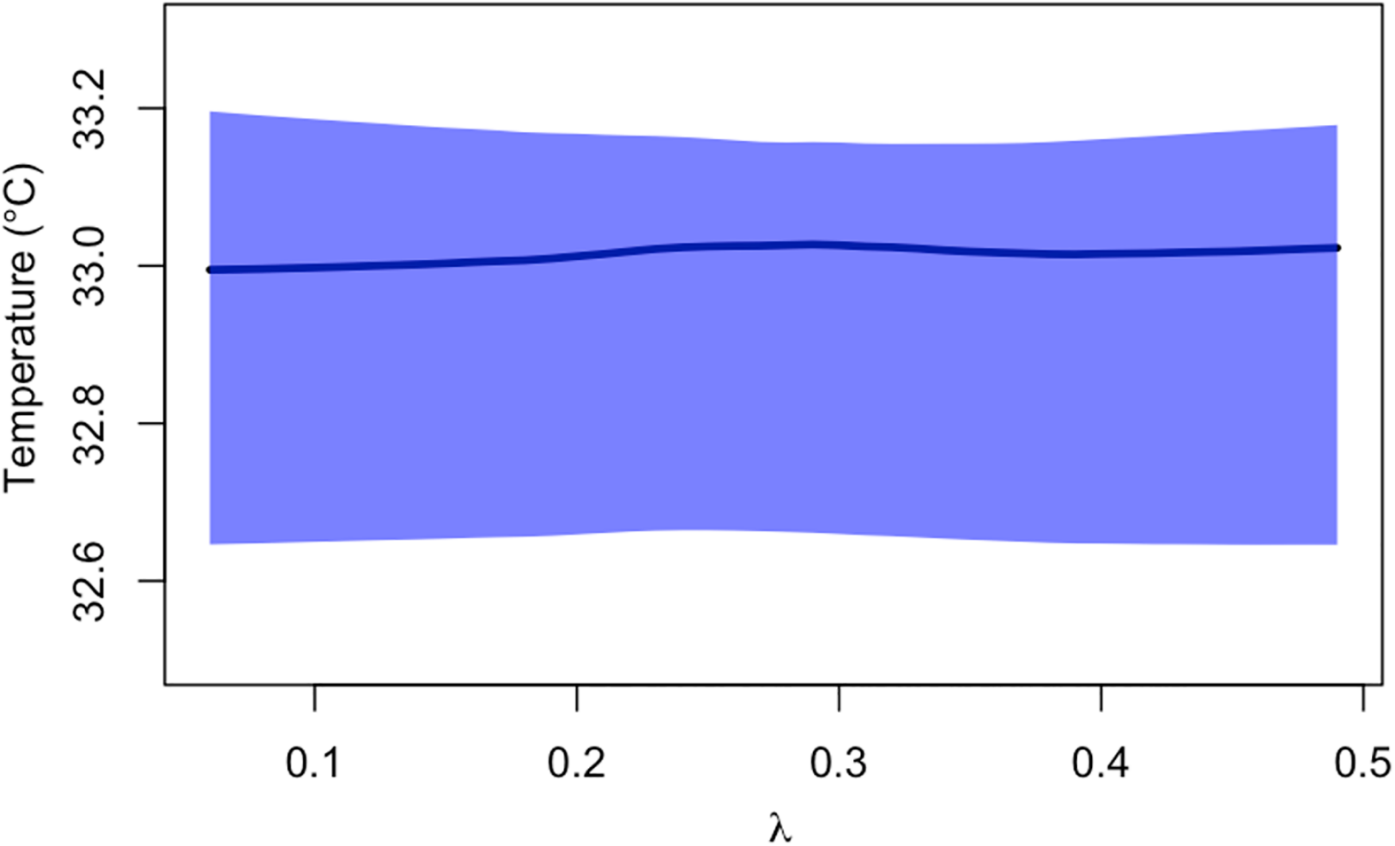
Temperature at which *r* peaks across a range of mosquito emergence rates λ, obtained by solving for *r* with 1,000 simulations of *R*_0_ based on Monte Carlo resampling of its three temperature-dependent parameters *μ*(*T*), *n*(*T*), and *a*(*T*) and applying Eq (1). The solid line is the median *r* at each λ value, and the shaded region shows the 95% confidence interval of *r* conditional on λ.

**Fig 4:**
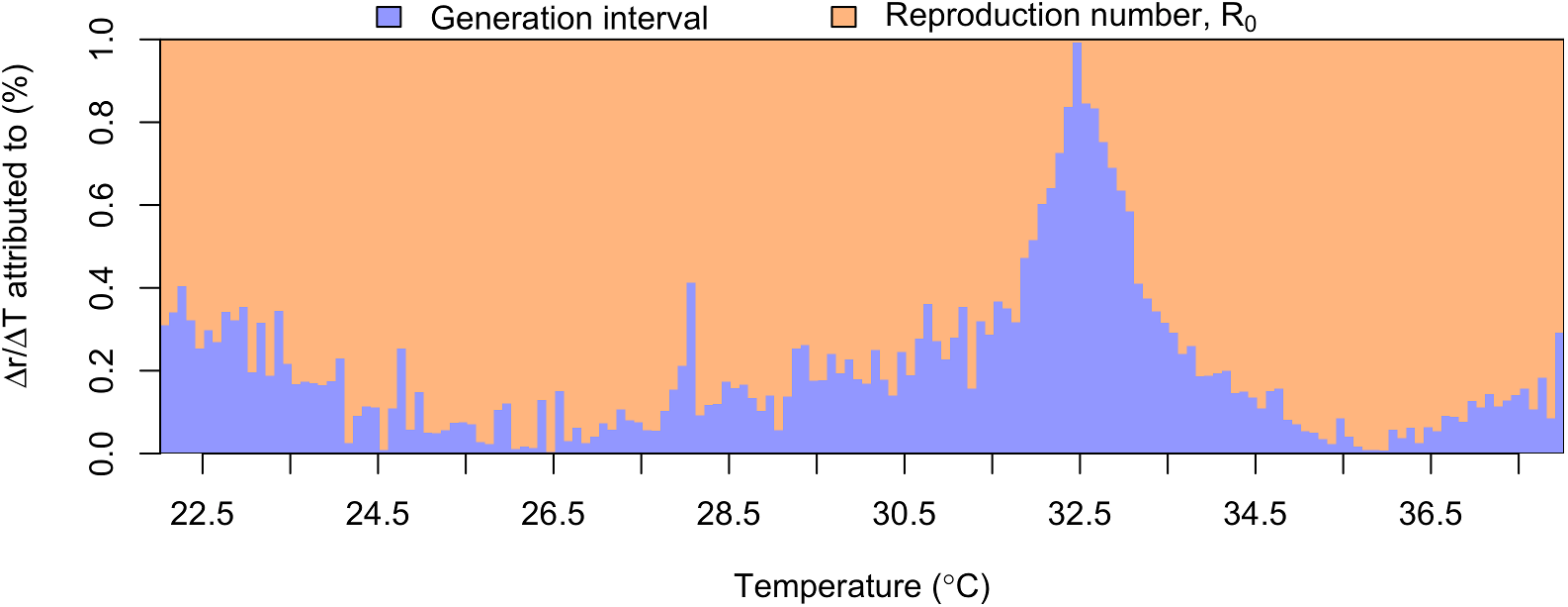
Relative contributions of the generation interval (blue) and the basic reproduction number *R*_0_ (orange) to temperature-driven changes in epidemic growth rate *r*. Temperature changes are considered in 0.1°C increments and assume λ = 0.2. See S1 Fig for consideration of alternative λ value.

Effects of temperature on the DENV generation interval and *R*_0_ contributed to similar effects on epidemic growth rate, *r*. Under mean estimates of model parameters, *r* increased with temperature until it peaked at 33°C (Fig 2D). Under 1,000 Monte Carlo samples of model parameters, peak temperature for *r* varied within a relatively narrow band with 95% of values falling between 32.6 and 33.2°C (Fig 3). As both *R*_0_ and the generation interval are temperature-dependent, changes in *r* due to temperature occur through both components. At a constant mosquito emergence rate *λ*, changes in *R*_0_ accounted for the majority of changes in *r*, although changes in the generation interval accounted for a greater degree of change near extreme and peak temperature regions (Fig 4; S1 Fig).

Allowing for diurnal temperature fluctuations (8°C daily temperature range for all mean temperature values) shortened the mean generation interval and increased its variance relative to a scenario with no diurnal temperature fluctuation (S2 Fig). Similarly, *R*_0_ decreased when DTR was considered, as the temperature at which *R*_0_ peaks decreased from 32.5 to 30.9°C due to the effect of daily temperature extrema (under DTR) on mosquito survival (S3 Fig). The combined effect of these changes on epidemic growth rate was a slight decrease, while the temperature at which the epidemic growth rate peaks remained close to its value under a scenario with no diurnal temperature fluctuation (Fig 3; S2–S4 Figs).

### How much detail is necessary to capture temperature effects on transmission?

**Fig 5:**
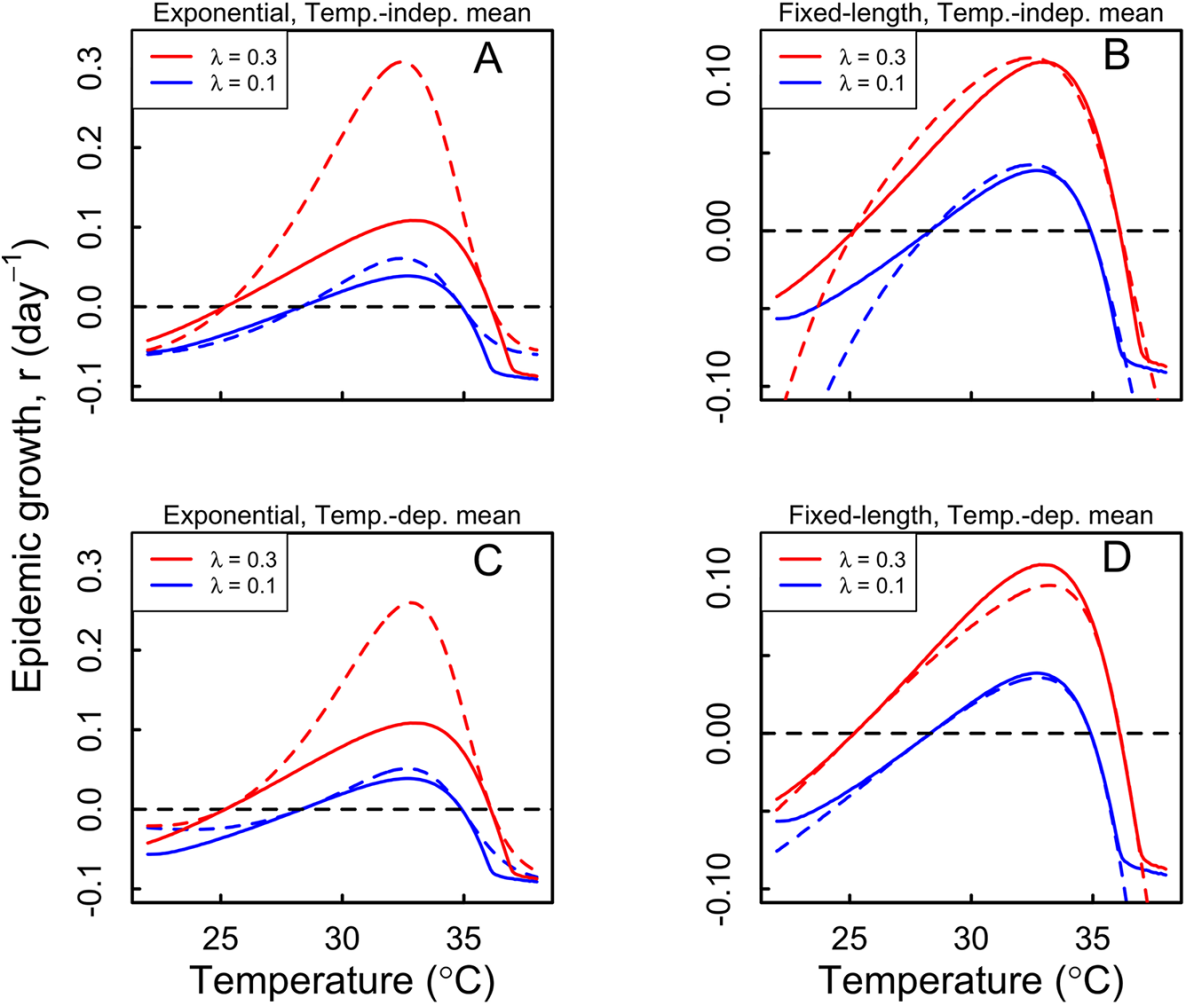
DENV epidemic growth rate, *r*, for high (red) and low (blue) mosquito densities based on our full model and other approximations. The top panels show comparisons of the full model estimates (solid lines) with those based on temperature independent, exponentially distributed (A) and fixed-length (B) generation intervals (mean = 16 days [34]) (dashed lines). The bottom panels show comparisons of estimates of the full model (solid lines) with those based on exponentially distributed (C) and fixed-length (D) generation intervals (dashed lines), with their mean values at each temperature set to the corresponding mean from the full model.

**Fig 6:**
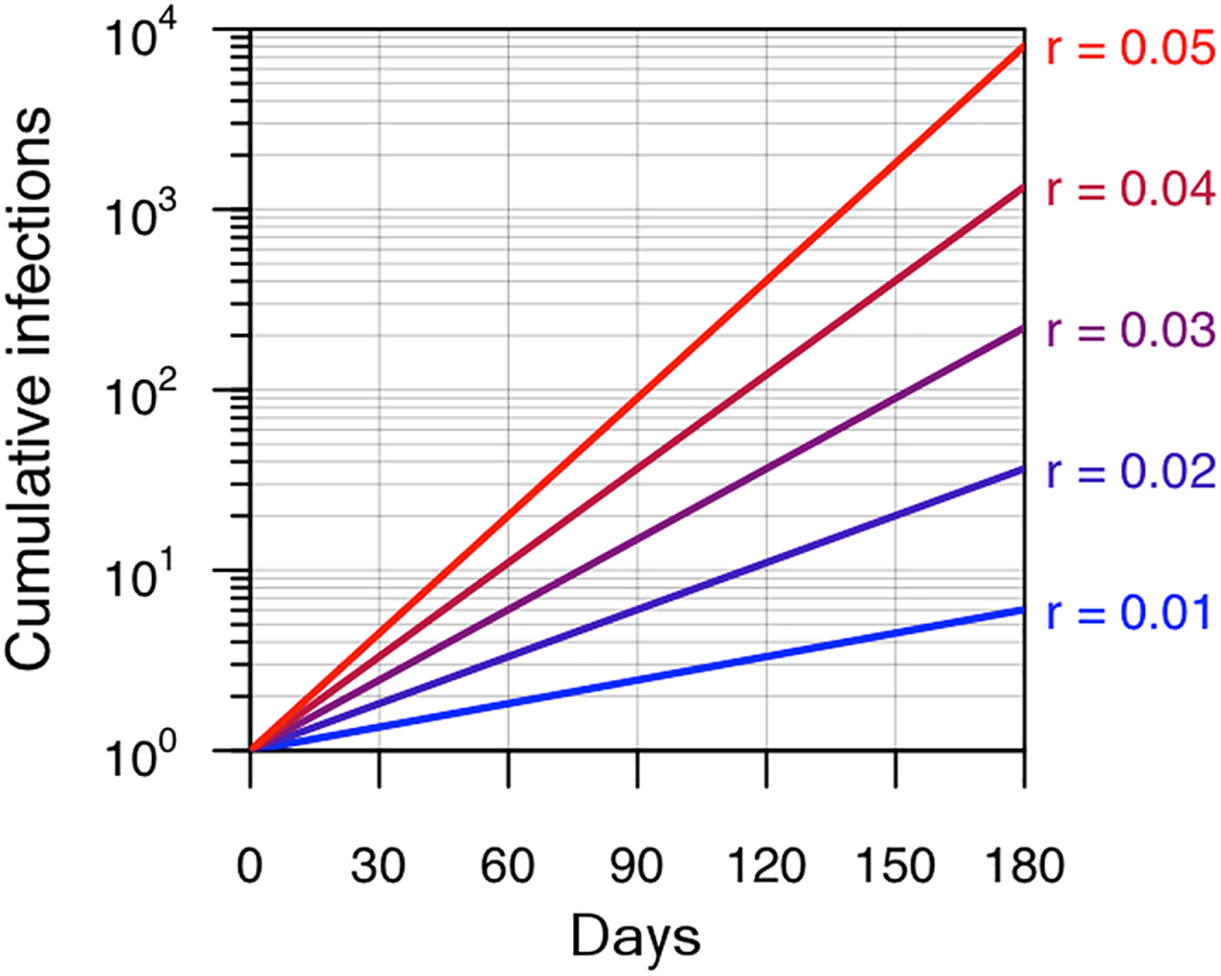
Epidemic growth under an exponential model with values of the epidemic growth rate *r* ranging from 0.01 to 0.05 for a duration of 180 days.

Because a fully detailed generation interval distribution is beyond the capabilities of many commonly used modeling frameworks [43], we examined the correspondence between epidemic growth rates *r* calculated under our detailed approach and under four less detailed approximations of the generation interval that are commonly used in transmission models. A fixed-length generation interval yielded a consistently better approximation of our detailed calculations of *r* as a function of temperature than did an exponentially distributed generation interval (Fig 5A vs. 5B). Calculations of *r* under the fixed-length approximation tended to match calculations of temperature-dependent *r* under the detailed generation interval distribution particularly well in temperature ranges of significance to epidemics (i.e., where *r* > 0) (Fig 5B). For both fixed-length and exponential generation interval distributions, allowing their mean values to follow the temperature-dependent model improved their correspondence with our detailed formulation of temperature-dependent *r* (Fig 5A & 5B vs. 5C & 5D). These differences in *r* resulting from different assumptions about the distribution and temperature dependence of *r* could be of significance to epidemic projections, given that differences in *r* as small as 0.01 can lead to differences in incidence projections of an order of magnitude only a few months into an epidemic (Fig 6).

### Putting temperature effects on transmission into context

Our result that the temperature threshold for maximum *r* was relatively constant around 33°C (95% CI: 32.6–33.2°C) offers a useful reference point. In a given area and with other factors held constant, an increase in temperatures beyond this threshold would imply first a rise and then a fall in *r* between present and future. An increase in temperatures that never exceeds this threshold would imply an increase in *r* between present and future. At most times of year in most regions of the world that are suitable for DENV transmission, temperature increases by 2050 are expected to fall into the latter category (i.e., remaining below 33°C), suggesting that temperature changes could increase epidemic growth rates in those areas (S1–S3 Tables, S5–S16 Figs). On the other hand, temperature increases by 2050 in regions such as India and the African Sahel are expected to exceed 33°C during April-June, potentially resulting in lower epidemic growth rates in those areas during a portion of the year (S1–S3 Tables, S5–S16 Figs).

## Discussion

The central advance that we have made is the development of a probabilistic description of the generation interval for dengue virus (DENV) that is based on first principles of transmission, synthesizes pertinent data for DENV and *Ae*. *aegypti*, and characterizes the generation interval as a function of temperature. Although there is little data with which to independently validate our calculations, the mode of our generation time distribution at optimal temperatures for transmission (approximately 16 days at 28–32°C) accords with independent estimates of this quantity based on statistical analyses of spatiotemporal dengue case data from Thailand (15–17 days) [34]. Combining this result with a temperature-dependent description of the basic reproduction number, *R*_0_, we obtained a temperature-dependent description of the epidemic growth rate, *r*. All of these quantities were estimated explicitly for DENV but are also relevant for other arboviruses such as chikungunya and Zika, given their similar ecology and given that many of the parameters we used are not specific to any one virus but instead to their common vector.

The generation interval has a wide range of applications in epidemiology, including the identification of sources of infection [44], the establishment of causal linkages between cases [45], and the characterization of temporal variation in transmission [8,46]. These and other studies have typically assumed a static generation interval of either fixed length [47] or with some standard statistical distribution [48]. Our result that the generation interval for DENV is not static but is instead highly dynamic with respect to temperature highlights that transmission models for DENV and other arboviruses could be systematically inaccurate by excluding temperature-dependent effects. Future work will be needed to address the existence and significance of any such inaccuracies, but our results about the sensitivity of *r* to the form of the generation interval and temperature dependency therein suggest that these effects could be substantial.

Our calculations of *R*_0_ are consistent with the notion that temperature plays an important role in determining optimal conditions for transmission (i.e., peak *R*_0_ at 32.5°C) and for delimiting conditions where transmission is sustainable (i.e., *R*_0_ > 1, Fig 2B and 2C). However, these results are only valid for a given value of the ratio of new adult mosquitoes to humans *λ*, which we allowed to vary within a plausible range due to the fact that it depends on a wide range of factors other than temperature. In particular, *λ* depends on the availability and quality of aquatic habitats for mosquitoes [49] and sociocultural factors that affect contact between people and mosquitoes [50]. Some studies have used temperature-based *R*_0_ calculations to delimit geographic ranges of other vector-borne diseases such as malaria [16], but we used *R*_0_ solely as part of an intermediate step to link the generation interval with epidemic growth rates.

Although *R*_0_ is important for quantifying threshold conditions for pathogen persistence, it is not well suited for characterizing temporal dynamics of transmission [51]. By combining temperature-dependent descriptions of *R*_0_ and the generation interval, our results offer a new way to characterize the intensity of dengue epidemics as a function of temperature. One common concern about analyses based on *R*_0_, and estimates of *r* based on *R*_0_, is whether they are relevant beyond the context of a novel pathogen in a fully susceptible population. Estimates of *r* based on the effective reproduction number, *R* [17], offer a more generalizable alternative to estimates of *r* based on *R*_0_, which is what we have considered in this study. To consider how the distinction between *R*_0_ and *R* might impact our results, we note the relationship *R* = *R*_0_*S*, where *S* is the proportion of a population that is susceptible. This linear relationship between *R*_0_ and *R* implies that extrapolating our results below *S* = 1 should result in behavior similar to how our temperature-dependent estimates of *r* vary with changes in *λ*, given that *λ* also affects *R*_0_ linearly. Perhaps most importantly, this reasoning implies that the temperature at which epidemic growth rates peak should be applicable across contexts in which either the susceptible fraction *S* or the mosquito-human ratio *λ* vary. Still other factors affecting *R*_0_ and *r* could vary across contexts—e.g., species or strain differences [52]—that could be important for some future applications.

One limitation of our approach is that the precise value of the temperature threshold for maximum *r* could be subject to revision as understanding of the relationships between temperature and transmission parameters improves. In previous work [15], revised assumptions about the effects of temperature on transmission parameters were shown to affect prior understanding of the relationship between temperature and *R*_0_ for malaria. Independently validating our calculations with epidemic data could be one way to address these uncertainties, but epidemic growth rates based on case reports can be difficult to compare across sites. Even if factors such as temperature are consistent across sites, still others may vary and have major impacts on epidemic growth rates, including mosquito abundance [39], population immunity [53], and reporting rates [54]. Due to these and other variations across locations, Johansson et al. [55] found no detectable association between temperature and large-scale epidemic dynamics. Our results make important progress towards being able to resolve the roles of and complex interactions among these factors in future studies.

Based on current understanding of relationships between temperature and transmission parameters, our result that *r* consistently peaks around 33°C (95% CI: 32.6–33.2°C) led us to examine which populations globally could remain below, newly exceed, or further surpass this temperature under future climate change scenarios. We found that most people currently living in areas at risk for DENV transmission could be subject to increased epidemic growth rates by 2050 under a range of scenarios about future temperature increases. For most DENV-endemic areas, this would have little effect on the overall burden of disease, which is already high, but it could affect transmission dynamics, making epidemics more intense. At the same time, there are a number of important caveats to bear in mind about these projections. First, transmission depends not only on temperature but also other abiotic variables, such as rainfall, in complex ways [56]. Second, the effects of abiotic variables may be outweighed by changes in human factors, such as economic development, urbanization, demography, and population immunity [57,58]. Third, long-term projections of dengue are highly variable and conflicting [59], making the long-term effects of any single change such as temperature nearly impossible to anticipate.

## Conclusion

Although *r* will vary across different regions for different reasons, our finding that temperature changes under future climate change could elevate epidemic intensity of dengue in some areas suggests a categorically new way in which climate change might impact infectious disease transmission [60]. Our quantification of these effects focused on DENV, but these results also offer tentative, but plausible, estimates of how epidemics of other viruses transmitted by *Ae*. *aegypti* mosquitoes, such as chikungunya and Zika, might be impacted under future climate change. Our qualitative results apply even more broadly, implying that temperature has the potential to shape multiple aspects of vector-borne parasite life history and to influence multiple aspects of the temporal dynamics of associated diseases.

## Supporting information

S1 Appendix Derivation of lifetime-averaged average biting rate. (PDF)

S2 Appendix Diurnally fluctuating hazards for mosquito mortality and EIP. (PDF)

S1 Fig Relative contributions the generation interval (blue) and the basic reproduction number *R*_0_ (orange) to temperature-driven changes in epidemic growth rate *r* under different values of *λ*. Temperature changes are considered in 0.1°C increments. Varying the mosquito emergence rate *λ* made little difference in the overall contribution of the generation interval and reproduction numbers. For lower values of *λ*, the contribution of the generation interval is more pronounced due to an overall lower *R*_0_. Values of λ considered here correspond to peak *R*_0_ values of 0.5, 1.0, 2.0, 4.0, 6.0, and 8.0, respectively.
(PDF)

S2 Fig DENV generation interval distributions at different temperatures assuming constant temperatures (solid line) and diurnal temperature fluctuations with a range of 8°C (dashed). (PDF)

S3 Fig Reproduction numbers as a function of temperature assuming different values of λ ranging 0.1–0.4. Solid lines represent values assuming constant temperatures, while dotted lines assume diurnal temperature fluctuations with a range of 8°C. The large drop in the reproduction number around 34°C is an artifact of the absolute maximum temperature thresholds that are exceeded when temperatures reach 37.73°C for consecutive three hours in a day. The reproduction number peaks at 32.5°C when constant temperature is assumed and at 30.9°C when diurnal fluctuation is assumed.
(PDF)

S4 Fig Epidemic growth rates as a function of temperature assuming different values of λ ranging 0.1–0.4. Solid lines represent values assuming constant temperatures, while dotted lines assume diurnal temperature fluctuations with a range of 8°C. The large drop in the reproduction number around 34°C is an artifact of the absolute maximum temperature thresholds that are exceeded when temperatures reach 37.73°C for consecutive three hours in a day. The epidemic growth rate peaks within the range 31.6–33.9°C.
(PDF)

S5 Fig Regions that fall into different categories with respect to their relationship to peak temperature of 33°C by 2050 in the month of January. These classifications are based on the RCP 4.5 temperature change scenario [36]. Regions are classified by color according to whether they are projected to remain below (red), newly exceed (yellow), or further surpass (green) the peak temperature of 33°C for maximizing epidemic growth rate, *r*. Gray areas are masked from this analysis due to their unsuitability for dengue transmission [39].
(PDF)

S6 Fig Regions that fall into different categories with respect to their relationship to peak temperature of 33°C by 2050 in the month of February. These classifications are based on the RCP 4.5 temperature change scenario [36]. Regions are classified by color according to whether they are projected to remain below (red), newly exceed (yellow), or further surpass (green) the peak temperature of 33°C for maximizing epidemic growth rate, *r*. Gray areas are masked from this analysis due to their unsuitability for dengue transmission [39].
(PDF)

S7 Fig Regions that fall into different categories with respect to their relationship to peak temperature of 33°C by 2050 in the month of March. These classifications are based on the RCP 4.5 temperature change scenario [36]. Regions are classified by color according to whether they are projected to remain below (red), newly exceed (yellow), or further surpass (green) the peak temperature of 33°C for maximizing epidemic growth rate, *r*. Gray areas are masked from this analysis due to their unsuitability for dengue transmission [39].
(PDF)

S8 Fig Regions that fall into different categories with respect to their relationship to peak temperature of 33°C by 2050 in the month of April. These classifications are based on the RCP 4.5 temperature change scenario [36]. Regions are classified by color according to whether they are projected to remain below (red), newly exceed (yellow), or further surpass (green) the peak temperature of 33°C for maximizing epidemic growth rate, *r*. Gray areas are masked from this analysis due to their unsuitability for dengue transmission [39].
(PDF)

S9 Fig Regions that fall into different categories with respect to their relationship to peak temperature of 33°C by 2050 in the month of May. These classifications are based on the RCP 4.5 temperature change scenario [36]. Regions are classified by color according to whether they are projected to remain below (red), newly exceed (yellow), or further surpass (green) the peak temperature of 33°C for maximizing epidemic growth rate, *r*. Gray areas are masked from this analysis due to their unsuitability for dengue transmission [39].
(PDF)

S10 Fig Regions that fall into different categories with respect to their relationship to peak temperature of 33°C by 2050 in the month of June. These classifications are based on the RCP 4.5 temperature change scenario [36]. Regions are classified by color according to whether they are projected to remain below (red), newly exceed (yellow), or further surpass (green) the peak temperature of 33°C for maximizing epidemic growth rate, *r*. Gray areas are masked from this analysis due to their unsuitability for dengue transmission [39].
(PDF)

S11 Fig Regions that fall into different categories with respect to their relationship to peak temperature of 33°C by 2050 in the month of July. These classifications are based on the RCP 4.5 temperature change scenario [36]. Regions are classified by color according to whether they are projected to remain below (red), newly exceed (yellow), or further surpass (green) the peak temperature of 33°C for maximizing epidemic growth rate, *r*. Gray areas are masked from this analysis due to their unsuitability for dengue transmission [39].
(PDF)

S12 Fig Regions that fall into different categories with respect to their relationship to peak temperature of 33°C by 2050 in the month of August. These classifications are based on the RCP 4.5 temperature change scenario [36]. Regions are classified by color according to whether they are projected to remain below (red), newly exceed (yellow), or further surpass (green) the peak temperature of 33°C for maximizing epidemic growth rate, *r*. Gray areas are masked from this analysis due to their unsuitability for dengue transmission [39].
(PDF)

S13 Fig Regions that fall into different categories with respect to their relationship to peak temperature of 33°C by 2050 in the month of September. These classifications are based on the RCP 4.5 temperature change scenario [36]. Regions are classified by color according to whether they are projected to remain below (red), newly exceed (yellow), or further surpass (green) the peak temperature of 33°C for maximizing epidemic growth rate, *r*. Gray areas are masked from this analysis due to their unsuitability for dengue transmission [39].
(PDF)

S14 Fig Regions that fall into different categories with respect to their relationship to peak temperature of 33°C by 2050 in the month of October. These classifications are based on the RCP 4.5 temperature change scenario [36]. Regions are classified by color according to whether they are projected to remain below (red), newly exceed (yellow), or further surpass (green) the peak temperature of 33°C for maximizing epidemic growth rate, *r*. Gray areas are masked from this analysis due to their unsuitability for dengue transmission [39].
(PDF)

S15 Fig Regions that fall into different categories with respect to their relationship to peak temperature of 33°C by 2050 in the month of November. These classifications are based on the RCP 4.5 temperature change scenario [36]. Regions are classified by color according to whether they are projected to remain below (red), newly exceed (yellow), or further surpass (green) the peak temperature of 33°C for maximizing epidemic growth rate, *r*. Gray areas are masked from this analysis due to their unsuitability for dengue transmission [39].
(PDF)

S16 Fig Regions that fall into different categories with respect to their relationship to peak temperature of 33°C by 2050 in the month of December. These classifications are based on the RCP 4.5 temperature change scenario [36]. Regions are classified by color according to whether they are projected to remain below (red), newly exceed (yellow), or further surpass (green) the peak temperature of 33°C for maximizing epidemic growth rate, *r*. Gray areas are masked from this analysis due to their unsuitability for dengue transmission [39].
(PDF)

S1 Table Total population globally that falls into different categories with respect to their relationship to peak temperature of 33°C by 2050 in each month. These projections are based on ensemble means of Global Circulation Models (GCMs) under three Representative Concentration Pathways (RCPs), climate change scenarios adopted by the International Panel for Climate Change (IPCC) [36].
(PDF)

S2 Table Total population globally that falls into different categories with respect to their relationship to the lower bound of the 95% CI of temperatures at which r peak (32.6°C) by 2050 in each month. These projections are based on ensemble means of Global Circulation Models (GCMs) under three Representative Concentration Pathways (RCPs), climate change scenarios adopted by the International Panel for Climate Change (IPCC) [36].
(PDF)

S3 Table Total population globally that falls into different categories with respect to their relationship to the upper bound of the 95% CI of temperatures at which r peak (33.2°C) by 2050 in each month. These projections are based on ensemble means of Global Circulation Models (GCMs) under three Representative Concentration Pathways (RCPs), climate change scenarios adopted by the International Panel for Climate Change (IPCC) [36].
(PDF)

S4 Table Model parameters and their default values. (PDF)
