# Supplementary material for: Temperature modulates dengue virus epidemic growth rates through its effects on reproduction numbers and generation intervals
Source: PLoS Negl Trop Dis. 2017 Jul 19;11(7):e0005797. doi: 10.1371/journal.pntd.0005797 (PMC5536440; doi:10.1371/journal.pntd.0005797)
Supplement: S1 Appendix — (PDF) [file pntd.0005797.s001.pdf]

S1 Appendix. Derivation of lifetime-averaged average biting rate

Here, we derive the average biting rate across a mosquito's lifetime when the rate a_1 at which it takes its first bite differs from the rate a_2 at which it takes subsequent bites. We assume that the time between bites is exponentially distributed, as is the mosquito's lifetime with death rate μ. First, we note that for a given age of first biting α and a given lifetime l, the average biting rate that applies over the course of the mosquito's life is

$$\frac{\alpha}{l}a_1 + \frac{l-\alpha}{l}a_2. \qquad \text{(S1)}$$

Second, for a given lifetime l, we calculate the expectation of the expression in (S1) by evaluating the integral

$$\int_0^l a_1 e^{-a_1\alpha}\left(\frac{\alpha}{l}a_1 + \frac{l-\alpha}{l}a_2\right) d\alpha, \qquad \text{(S2)}$$

which equals

$$\frac{e^{-a_1 l}\left(e^{a_1 l}(a_1 a_2 l + a_1 - a_2) - a_1(a_1 l + 1) + a_2\right)}{a_1 l\left(1 - e^{-a_1 l}\right)}. \qquad \text{(S3)}$$

Finally, we solve for the average biting rate $\bar{a}$ across a mosquito's lifetime as a function of mosquito death rate μ and the two component biting rates a_1 and a_2 as

$$\bar{a}(\mu, a_1, a_2) = \int_0^\infty \mu e^{-\mu l}\left(\frac{e^{-a_1 l}\left(e^{a_1 l}(a_1 a_2 l + a_1 - a_2) - a_1(a_1 l + 1) + a_2\right)}{a_1 l\left(1 - e^{-a_1 l}\right)}\right) dl. \quad \text{(S4)}$$

The integral in eq. (S4) is not practical to solve analytically, so we solved it numerically using the integrate function in R [1] when calculating $\bar{a}$ in our analyses. We also note that μ, a_1, and a_2 are all functions of temperature, meaning that so too is $\bar{a}$.

Reference

1. R Core Team. 2016. R: A language and environment for statistical computing.
