# Supplementary material for: Temperature modulates dengue virus epidemic growth rates through its effects on reproduction numbers and generation intervals
Source: PLoS Negl Trop Dis. 2017 Jul 19;11(7):e0005797. doi: 10.1371/journal.pntd.0005797 (PMC5536440; doi:10.1371/journal.pntd.0005797)
Supplement: S2 Appendix — (PDF) [file pntd.0005797.s002.pdf]

S2 Appendix. Diurnally fluctuating hazards for mosquito mortality and EIP.

With temperature fluctuating diurnally [10,41], we considered a time-varying mosquito survivorship hazard corresponding to time-varying temperatures. We began by first generating temperature-dependent mortality rates $\Lambda(T)$ based on diurnally fluctuating temperature profiles $T(t)$. To translate these rates into time-varying hazards $h(t)$ (defined as the conditional probability of an event happening within a short time Δt given that it has not yet happened), we applied

$$h(t) = 1 - \exp\left(-\Lambda\left(T(t)\right) \Delta t\right), \qquad \text{(S5)}$$

where Δt is the smallest time increment we considered. We then applied these hazards iteratively to a survivorship function $S(t)$ (defined as the complement of the distribution function $F(t)$ of the random variable describing the timing of death) beginning at $S(0) = 1$ and updating

$$S(t + \Delta t) = S(t)\left(1 - h(t + \Delta t)\right) \qquad \text{(S6)}$$

for each Δt. Obtaining the probability density associated with the random variable in question was achieved by first calculating $F(t) = 1 - S(t)$ and then differentiating $F(t)$ with respect to t to obtain $f(t)$. This same process was applied to capture time-varying effects of temperature on mortality and EIP by substituting $\Lambda(T)$ for $\mu(T)$ and 1/EIP(T), respectively.
