# Supplementary material for: Temperature modulates dengue virus epidemic growth rates through its effects on reproduction numbers and generation intervals
Source: PLoS Negl Trop Dis. 2017 Jul 19;11(7):e0005797. doi: 10.1371/journal.pntd.0005797 (PMC5536440; doi:10.1371/journal.pntd.0005797)
Supplement: S1 Fig — Temperature changes are considered in 0.1°C increments. Varying the mosquito emergence rate λ made little difference in the overall contribution of the generation interval and reproduction numbers. For lower values of λ, the contribution of the generation interval is more pronounced due to an overall lower R0. Values of λ considered here correspond to peak R0 values of 0.5, 1.0, 2.0, 4.0, 6.0, and 8.0, respectively. (PDF) [file pntd.0005797.s003.pdf]

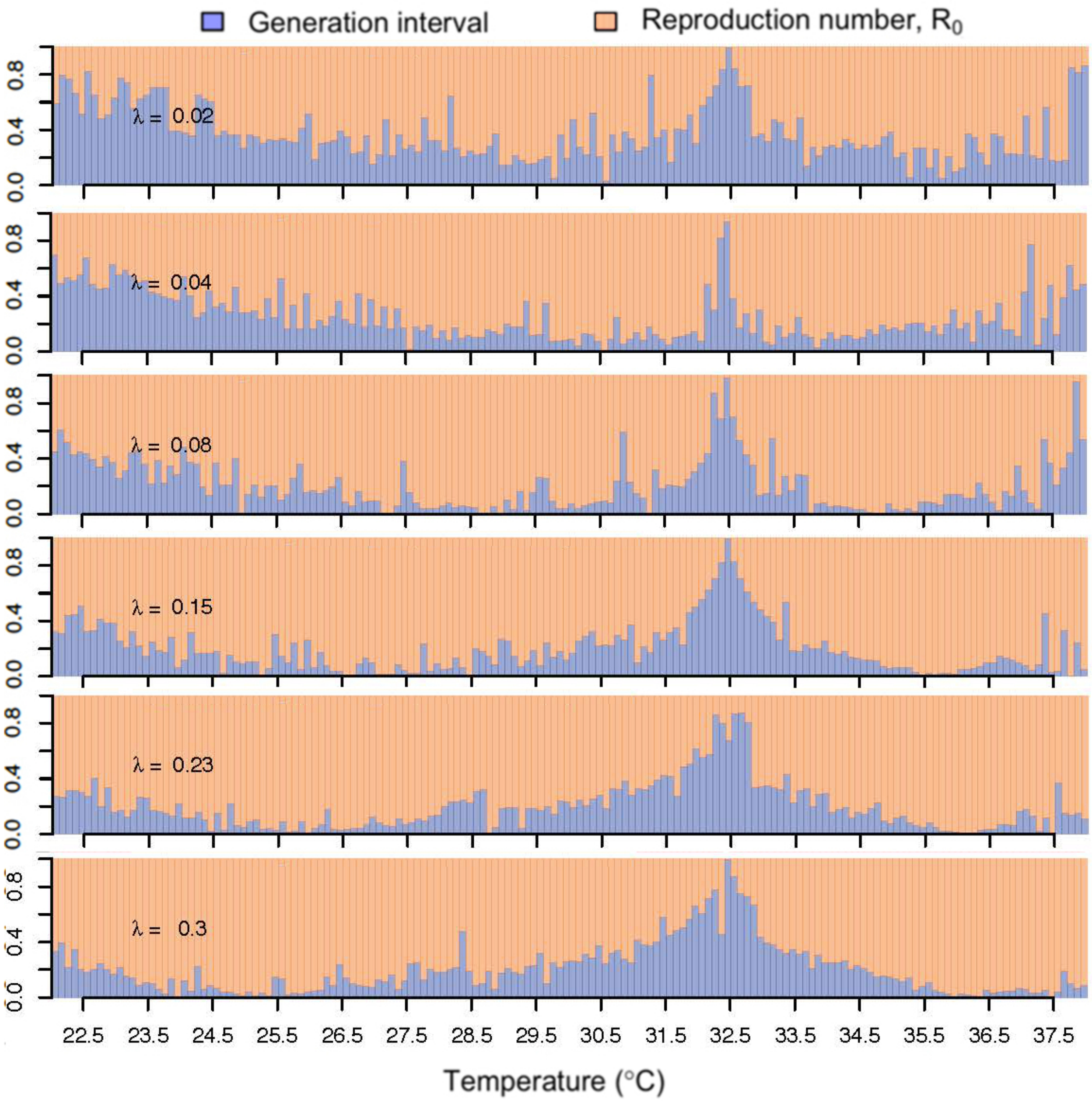

S1 Figure. **Relative contributions the generation interval (blue) and the basic reproduction number R_0 (orange) to temperature-driven changes in epidemic growth rate *r* under different values of λ.** Temperature changes are considered in 0.1 °C increments. Varying the mosquito emergence rate λ made little difference in the overall contribution of the generation interval and reproduction numbers. For lower values of λ, the contribution of the generation interval is more pronounced due to an overall lower R_0. Values of λ considered here correspond to peak R_0 values of 0.5, 1.0, 2.0, 4.0, 6.0, and 8.0, respectively.
