# Supplementary material for: Temperature modulates dengue virus epidemic growth rates through its effects on reproduction numbers and generation intervals
Source: PLoS Negl Trop Dis. 2017 Jul 19;11(7):e0005797. doi: 10.1371/journal.pntd.0005797 (PMC5536440; doi:10.1371/journal.pntd.0005797)
Supplement: S2 Fig — (PDF) [file pntd.0005797.s004.pdf]

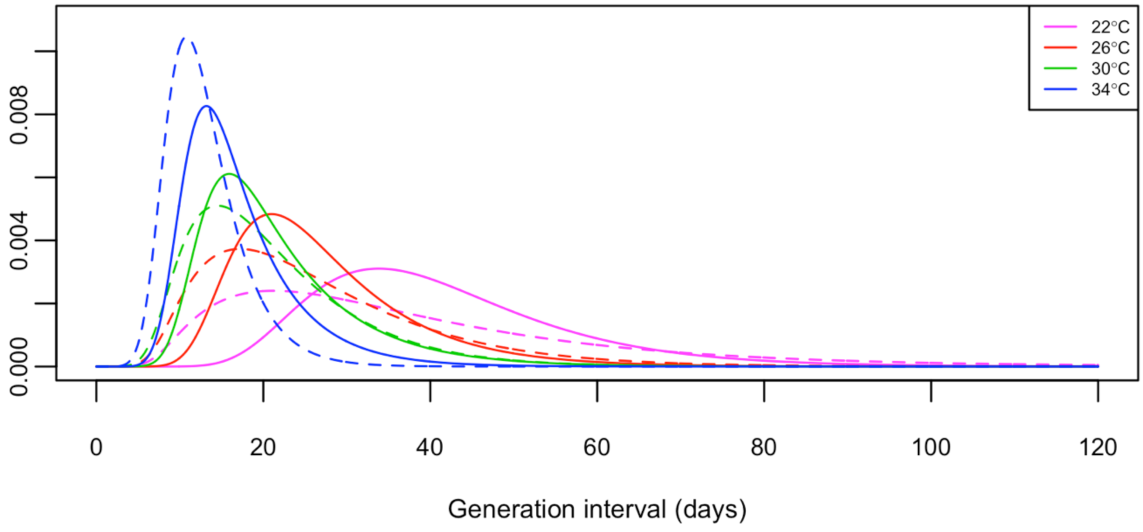

S2 Figure. DENV generation interval distributions at different temperatures assuming constant temperatures (solid line) and diurnal temperature fluctuations with a range of 8 °C (dashed).
