# Supplementary material for: Temperature modulates dengue virus epidemic growth rates through its effects on reproduction numbers and generation intervals
Source: PLoS Negl Trop Dis. 2017 Jul 19;11(7):e0005797. doi: 10.1371/journal.pntd.0005797 (PMC5536440; doi:10.1371/journal.pntd.0005797)
Supplement: S3 Fig — Solid lines represent values assuming constant temperatures, while dotted lines assume diurnal temperature fluctuations with a range of 8°C. The large drop in the reproduction number around 34°C is an artifact of the absolute maximum temperature thresholds that are exceeded when temperatures reach 37.73°C for consecutive three hours in a day. The reproduction number peaks at 32.5°C when constant temperature is assumed and at 30.9°C when diurnal fluctuation is assumed. (PDF) [file pntd.0005797.s005.pdf]

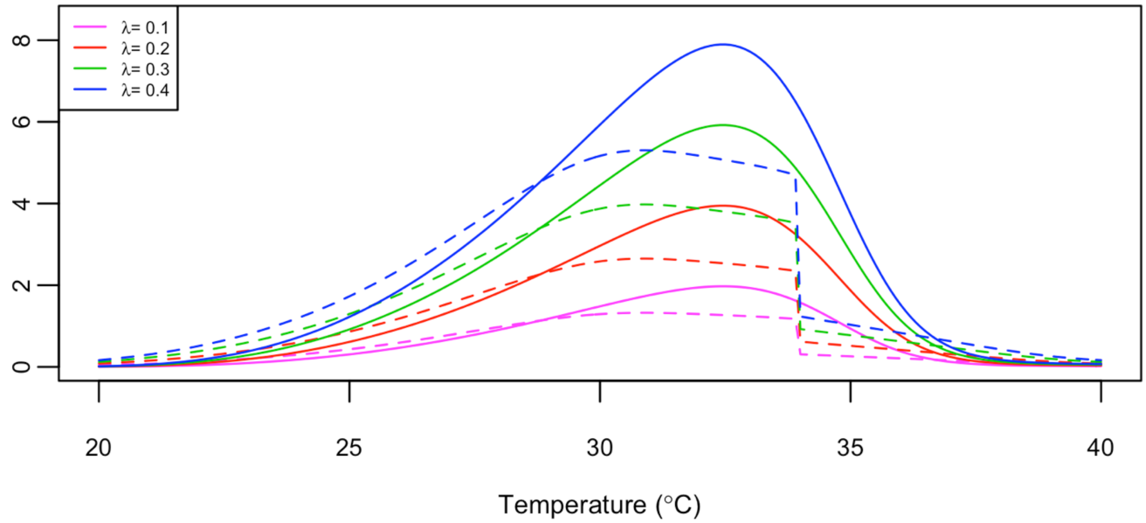


**S3 Figure. Reproduction numbers as a function of temperature assuming different values of λ ranging 0.1-0.4.** Solid lines represent values assuming constant temperatures, while dotted lines assume diurnal temperature fluctuations with a range of 8 °C. The large drop in the reproduction number around 34 °C is an artifact of the absolute maximum temperature thresholds that are exceeded when temperatures reach 37.73 °C for consecutive three hours in a day. The reproduction number peaks at 32.5 °C when constant temperature is assumed and at 30.9 °C when diurnal fluctuation is assumed.
