# Supplementary material for: Temperature modulates dengue virus epidemic growth rates through its effects on reproduction numbers and generation intervals
Source: PLoS Negl Trop Dis. 2017 Jul 19;11(7):e0005797. doi: 10.1371/journal.pntd.0005797 (PMC5536440; doi:10.1371/journal.pntd.0005797)
Supplement: S4 Fig — Solid lines represent values assuming constant temperatures, while dotted lines assume diurnal temperature fluctuations with a range of 8°C. The large drop in the reproduction number around 34°C is an artifact of the absolute maximum temperature thresholds that are exceeded when temperatures reach 37.73°C for consecutive three hours in a day. The epidemic growth rate peaks within the range 31.6–33.9°C. (PDF) [file pntd.0005797.s006.pdf]

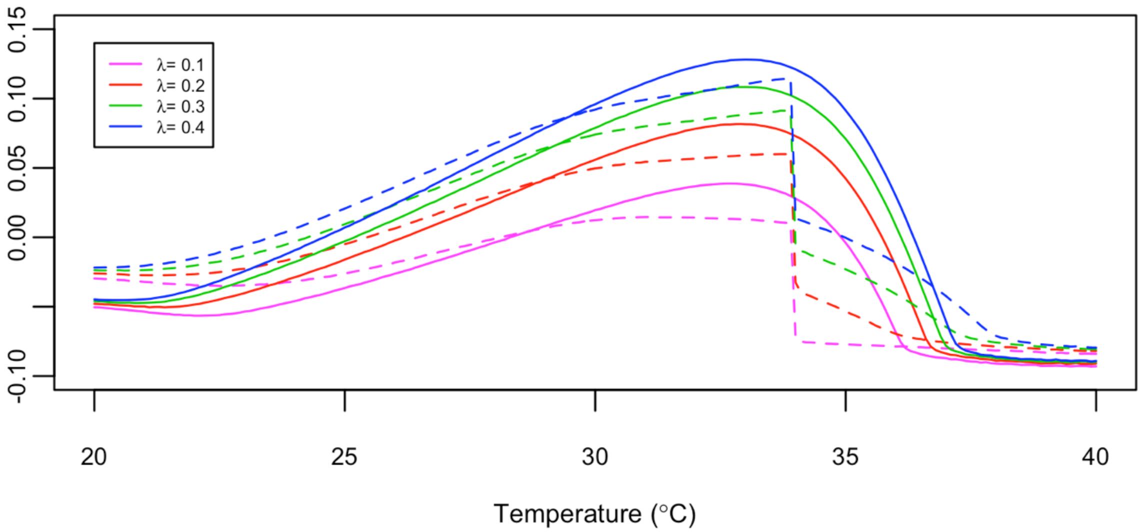

S4 Figure. Epidemic growth rates as a function of temperature assuming different values of λ ranging 0.1-0.4. Solid lines represent values assuming constant temperatures, while dotted lines assume diurnal temperature fluctuations with a range of 8 °C. The large drop in the reproduction number around 34 °C is an artifact of the absolute maximum temperature thresholds that are exceeded when temperatures reach 37.73 °C for consecutive three hours in a day. The epidemic growth rate peaks within the range 31.6-33.9 °C.
