# Supplementary material for: Temperature modulates dengue virus epidemic growth rates through its effects on reproduction numbers and generation intervals
Source: PLoS Negl Trop Dis. 2017 Jul 19;11(7):e0005797. doi: 10.1371/journal.pntd.0005797 (PMC5536440; doi:10.1371/journal.pntd.0005797)
Supplement: S9 Fig — These classifications are based on the RCP 4.5 temperature change scenario [36]. Regions are classified by color according to whether they are projected to remain below (red), newly exceed (yellow), or further surpass (green) the peak temperature of 33°C for maximizing epidemic growth rate, r. Gray areas are masked from this analysis due to their unsuitability for dengue transmission [39]. (PDF) [file pntd.0005797.s011.pdf]

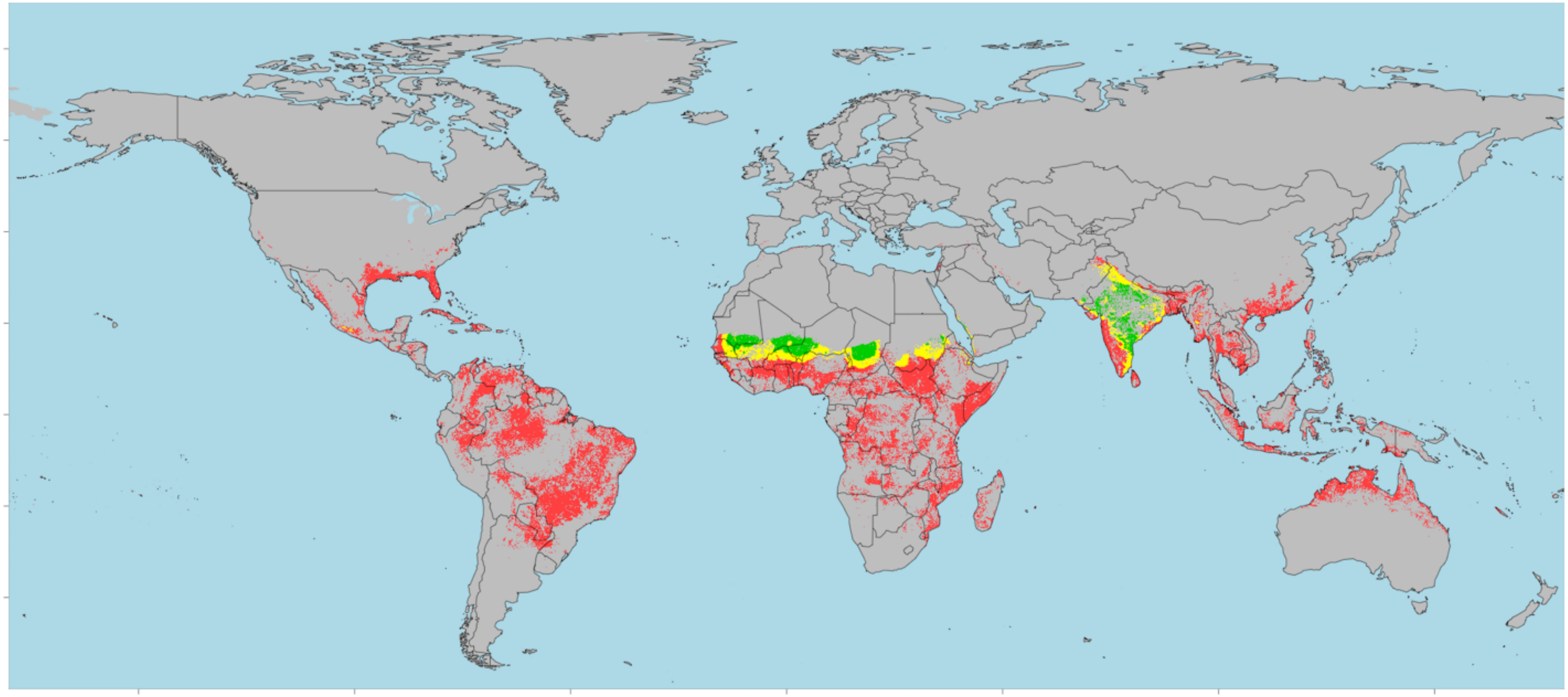

S9 Figure. Regions that fall into different categories with respect to their relationship to peak temperature of 33 °C by 2050 in the month of May. These classifications are based on the RCP 4.5 temperature change scenario [36]. Regions are classified by color according to whether they are projected to remain below (red), newly exceed (yellow), or further surpass (green) the peak temperature of 33 °C for maximizing epidemic growth rate, *r*. Gray areas are masked from this analysis due to their unsuitability for dengue transmission [39].
