# Supplementary material for: Temperature modulates dengue virus epidemic growth rates through its effects on reproduction numbers and generation intervals
Source: PLoS Negl Trop Dis. 2017 Jul 19;11(7):e0005797. doi: 10.1371/journal.pntd.0005797 (PMC5536440; doi:10.1371/journal.pntd.0005797)
Supplement: S3 Table — These projections are based on ensemble means of Global Circulation Models (GCMs) under three Representative Concentration Pathways (RCPs), climate change scenarios adopted by the International Panel for Climate Change (IPCC) [36]. (PDF) [file pntd.0005797.s021.pdf]

S3 Table. Total population globally that falls into different categories with respect to their relationship to the upper bound of the 95% CI of temperatures at which *r* peak (33.2 °C) by 2050 in each month. These projections are based on ensemble means of Global Circulation Models (GCMs) under three Representative Concentration Pathways (RCPs), climate change scenarios adopted by the International Panel for Climate Change (IPCC) [36].

	RCP 4.5			RCP 6.0			RCP 8.5		
Month	Remain	Newly	Further	Remain	Newly	Further	Remain	Newly	Further
Jan.	3,196.9	0.0	0.0	3,196.9	0.0	0.0	3,196.9	0.0	0.0
Feb.	3,196.7	0.2	0.0	3,196.7	0.2	0.0	3,196.6	0.3	0.0
Mar.	3,158.7	38.2	0.1	3,174.2	22.6	0.1	3,110.0	86.8	0.1
Apr.	2,819.0	356.8	21.0	2,918.7	257.2	21.0	2,662.4	513.4	21.0
May	2,293.3	541.2	362.4	2,358.9	475.6	362.4	2,217.0	617.5	362.4
Jun.	2,534.8	360.5	301.6	2,574.0	321.3	301.6	2,452.9	442.4	301.6
Jul.	3,092.0	94.1	10.8	3,120.6	65.5	10.8	3,065.5	120.6	10.8
Aug.	3,164.9	27.7	4.3	3,177.0	15.6	4.3	3,148.9	43.7	4.3
Sep.	3,181.3	14.6	1.0	3,186.1	9.7	1.0	3,169.0	26.9	1.0
Oct.	3,185.4	11.5	0.0	3,187.6	9.3	0.0	3,168.6	28.3	0.0
Nov.	3,196.9	0.0	0.0	3,196.9	0.0	0.0	3,196.9	0.0	0.0
Dec.	3,196.9	0.0	0.0	3,196.9	0.0	0.0	3,196.9	0.0	0.0
