# Supplementary material for: Temperature modulates dengue virus epidemic growth rates through its effects on reproduction numbers and generation intervals
Source: PLoS Negl Trop Dis. 2017 Jul 19;11(7):e0005797. doi: 10.1371/journal.pntd.0005797 (PMC5536440; doi:10.1371/journal.pntd.0005797)
Supplement: S4 Table — (PDF) [file pntd.0005797.s022.pdf]

S4 Table. Model parameters and their default values.

Parameter	Default value	Source
IIP distribution	lognormal(1.75, 0.27)	Chan and Johansson [12]
EIP distribution, $n(T)$	lognormal(exp(2.9 - 0.08T), 0.451)	Chan and Johansson [12]
Human-to-mosquito transmission period (HMTP)	Normal(3.18,1.15) + 2	Nishiura and Halstead [22]
Lab-based mortality rate, $\mu_L(T)$	generalized additive model with a smooth spline term for temperature	Brady et al. [11]
Field correction term, ρ (where T_1=20 °C, T_n=34 °C)	$\frac{1}{n}\sum_{T=T_1}^{T_n}(0.115 - \mu_L(T))$	Muir and Kay [21]
Mosquito mortality in the field, $\mu(T)$	$\mu_L(T) + \rho$	Derived
Average mosquito lifespan, $l(T)$	$1 / \mu(T)$	Derived
First biting rate, $a_1(T)$	$a_1(T)$	Focks et al. [9]
Second biting rate, $a_2(T)$	$a_2(T)$	Otero et al. [31]
Infectiousness of mosquito to human, b	0.4	Nishiura [30]
Human infectious period (days) weighted by infectiousness of human to mosquito	3.5	Nishiura and Halstead [22]
